# The Sound of Silence: Activating Silent Biosynthetic Gene Clusters in Marine Microorganisms

**DOI:** 10.3390/md13084754

**Published:** 2015-07-31

**Authors:** F. Jerry Reen, Stefano Romano, Alan D.W. Dobson, Fergal O’Gara

**Affiliations:** 1 BIOMERIT Research Centre, School of Microbiology, University College Cork—National University of Ireland, Cork, Ireland; E-Mails: j.reen@ucc.ie (F.J.R.); stefano.romano@ucc.ie (S.R.); 2 School of Microbiology, University College Cork—National University of Ireland, Cork, Ireland; E-Mail: a.dobson@ucc.ie; 3 School of Biomedical Sciences, Curtin University, Perth WA 6845, Australia

**Keywords:** silent, cryptic, bioactive gene clusters, polyketide synthases, non-ribosomal peptide synthases, metagenomics, heterologous expression

## Abstract

Unlocking the rich harvest of marine microbial ecosystems has the potential to both safeguard the existence of our species for the future, while also presenting significant lifestyle benefits for commercial gain. However, while significant advances have been made in the field of marine biodiscovery, leading to the introduction of new classes of therapeutics for clinical medicine, cosmetics and industrial products, much of what this natural ecosystem has to offer is locked in, and essentially hidden from our screening methods. Releasing this silent potential represents a significant technological challenge, the key to which is a comprehensive understanding of what controls these systems. Heterologous expression systems have been successful in awakening a number of these cryptic marine biosynthetic gene clusters (BGCs). However, this approach is limited by the typically large size of the encoding sequences. More recently, focus has shifted to the regulatory proteins associated with each BGC, many of which are signal responsive raising the possibility of exogenous activation. Abundant among these are the LysR-type family of transcriptional regulators, which are known to control production of microbial aromatic systems. Although the environmental signals that activate these regulatory systems remain unknown, it offers the exciting possibility of evoking mimic molecules and synthetic expression systems to drive production of potentially novel natural products in microorganisms. Success in this field has the potential to provide a quantum leap forward in medical and industrial bio-product development. To achieve these new endpoints, it is clear that the integrated efforts of bioinformaticians and natural product chemists will be required as we strive to uncover new and potentially unique structures from silent or cryptic marine gene clusters.

## 1. Introduction

The realization that microorganisms produce a vast array of diverse and functionally attractive compounds has underpinned medical and industrial developments over the decades. As recent as 2012, for small-molecule pharmaceuticals, 68% of the anticancer agents and 52% of the anti-infective agents are natural products, or derived from natural products [1]. Indeed, natural products are known to reflect the diversity and richness of our natural ecosystems, honed through infinite stages of evolutionary development. The diversity of producing organisms is probably still underestimated, with current bioactive microbial products, *i.e.*, antifungal, antibacterial, antiviral, cytotoxic and immunosuppressive agents, being isolated primarily from the fungal kingdom (*ca.* 42%), followed by strains belonging to the genus *Streptomyces* (32.1%) [2,3]. The culturable bottleneck that exists with the isolation and cultivation of marine isolates supports the expectation of a currently untapped reservoir of producing organisms. However, this diversity brings with it its own challenges whereby partly understood living organisms replace defined synthetic systems as the source of these potential drug products. While chemical synthesis programmes, challenging as they are, provide stepwise closed system production pipelines, isolation and production of secondary metabolites from living organisms requires detailed understanding of the physiology of the producing species, something that may not be readily available for newly isolated marine bacteria.

The biosynthetic capacity for small molecule production more often than not resides within discrete localized sections of the microbial genome, hence the term “biosynthetic gene clusters” (BGCs). Different classes exist, categorized based on the type of small molecular entity produced, with nonribosomal peptide synthetases (NRPSs), polyketides (PKs), ribosomally synthesized and post-translationally modified peptides (RiPPs), terpenoids, saccharides, and hybrid compounds being the most widely characterized. Typically exhibiting a characteristic modularity of enzymatic domains, BGCs can exceed 100 kb in size, a feature that creates significant bottlenecks in the use of molecular biology approaches to capture their activities [4]. Evolutionary insights into how nature can evolve new natural products have provided a basis upon which to interpret the diversity of existing BGCs [5]. While the diversity of chemical structures produced by BGCs is as vast as the biological activities they exhibit, the core enzymology used to biosynthesize these natural products is conserved. Many BGCs consist of repeating units or modules typically controlling the incorporation of one molecular entity into the final structure. As a result, the structural diversity of, e.g., the NRPS-synthesized natural products comes from variations in the number and order of the modules or amino acids incorporated [6]. Notwithstanding this, early predictions of a mix and match natural combinatorial strategy have not yet been realized, with active clusters exhibiting an exquisite level of conserved functionality.

**Figure 1:**
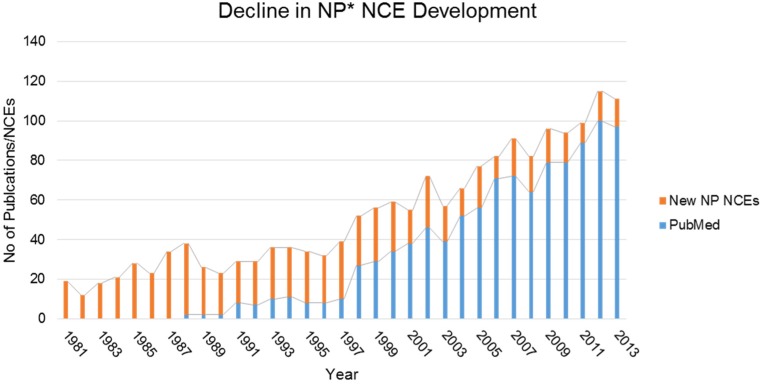
The decline in new chemical entities (NCE) development has continued in spite of an exponential increase in biosynthetic gene clusters (BGC) discovery. There has been more than a 50% reduction in the number of NCEs being approved by the FDA which can be categorized as natural products or derived from such. This has occurred at the same time that an explosion of papers citing “biosynthetic gene clusters” has emerged in the literature. Notwithstanding the lag time in drug development, serious bottlenecks exist in translating the bioactive potential from these microbial systems.

The traditional approach used for the bioassay-guided discovery of secondary metabolites, or natural products, has been based on the cultivation of microorganisms, chemical extraction of the produced metabolites, and final structure elucidation. Although this work-flow allowed the discovery of many valuable chemicals, nowadays it leads too often to rediscovery of known metabolites, leading to a dramatic reduction in the number of new molecules described [7]. A consequence of this is the declining number of new chemical entities (NCEs) in the drug development pipeline that have been observed in recent years (Figure 1), recently reviewed by both Gaudêncio & Pereiraa and Patridge and colleagues [8,9]. Having already identified >2000 antibiotics after screening of ≥10 million microbes, there is a clear realization that, following traditional approaches, the antibiotics yet to be discovered are likely to be produced by microbes less abundant than those that have already been discovered [10]. If this is true, and taking actinomycetes as an example, that means that new antibiotic producing actinomycetes will be picked at frequencies ≤10^−7^ per random isolate. Finding the precious few will require the screening of tens of millions of actinomycetes, while excluding or rapidly dereplicating the known antibiotics [10]. To counteract the pending stasis in novel product discovery, new approaches are needed to unlock the full potential of the “microgenome”. A key driver of this will be unlocking the silent potential of the microbial world, activating those systems that heretofore have remained locked away within the complex communities in which they are naturally produced.

In some cases, simply culturing a new organism will unlock novel bioactive compounds as seen with the isolation of the arugosins from the marine-derived fungus *Emericella nidulans* var. *acristata* or deoxypodophyllotoxin, a pro-drug of the anticancer drug podophyllotoxin, in the endophytic fungus *Aspergillus fumigatus* Fresenius [11,12]. However, even with the best advances in culturing technologies, there remains a significant untapped resource within microbial genomes, as evidenced by the advent of high throughput sequencing technology. These data revealed that the biosynthetic abilities of even the most well characterized organisms has been greatly unexplored [13,14]. In fact, the number of biosynthetic genes in many bacteria and fungi greatly outnumbers the known metabolites described for these organisms. This has led to the term “silent” or “cryptic” genes being phrased.

For example, the model fungus *A. nidulans* is potentially able to produce 32 polyketides, 14 non-ribosomal peptides and two indole alkaloids [15,16], with little more than 50% of the produced secondary metabolites being yet identified. There are several reasons for such an observation. In the first place it could be that, under laboratory cultivation conditions, the metabolites are produced in such an amount that cannot be easily detected. On the other hand, it is possible that the biosynthetic genes are not expressed, because their activation relies on environmental cues missing in laboratory conditions. Even if weakly expressed, the presence of toxic or PAIN (pan-assay interference) compounds may mask the effects of the low abundance compounds in question, making them more difficult to detect in the sea of secondary metabolites, while high throughput technologies have thus far failed to keep pace with the requirements of drug development pipelines. Furthermore, while much focus has centered on the production of specific compounds from defined pathway, it has also become clear that many compounds can emerge from a single pathway, depending on the culture conditions and the mechanisms used to elicit activation [17,18].

**Figure 2:**
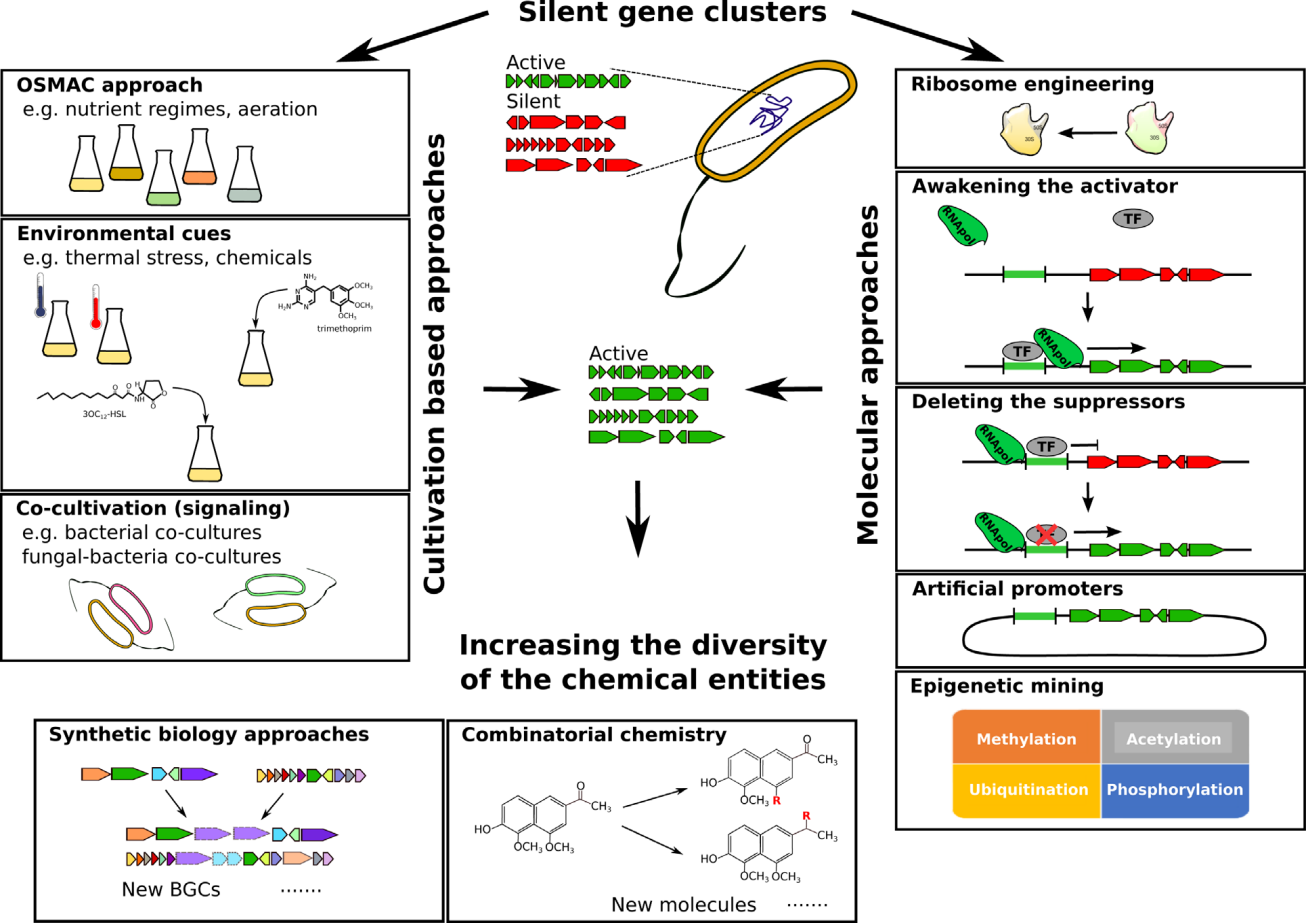
The integration of classical culture based and molecular biology based approaches for the elicitation of natural products from silent BGCs. A major challenge to the biodiscovery community, the cooperation of biological and chemical expertise is driving integrative technology developments that will unlock the silent potential of the cryptic gene clusters. Synthetic biology and combinatorial chemistry approaches further underpin the potential diversity of natural product that can be achieved.

In some ways, the silence of marine BGCs is analogous to the deafening quiet on standard culture media plates when marine samples are harvested. Our inability to cultivate the marine microflora, and particularly the low abundant producers of potentially novel natural products, feeds into the bottleneck in the pipeline of natural product discovery. Technology developments in the field of marine microbial cultivation will have significant application in the area of natural product research, and particularly with reference to the activation of silent biosynthetic clusters. Advances in our understanding of nutritional cues, signal based cell–cell communication, and synergistic interactions will feed into the toolkit for cryptic BGC activation. While the identification of silent gene clusters has already expanded our consideration of the biosynthetic ability of microorganisms, the development of multiple approaches to stimulate the production of unknown secondary metabolites remains a key challenge in harnessing the value of these cryptic systems (Figure 2). This review aims to present an overview on the most successful approaches adopted so far, focusing on the unexplored potential hidden in marine microorganisms.

## 2. Environmental Cues and Co-Cultivation

### 2.1. Stimulating Secondary Metabolite Production through Changing Culture Conditions: The “OSMAC” Approach

Microbial physiology is influenced by cultivation environment, media and conditions [19]. It follows, therefore, that the production of secondary metabolites and bioactive compounds will be influenced by metabolic factors, themselves influenced by the prevailing environment. Broadly considered a form of strain improvement, manipulation of culture conditions has been used for decades as a mechanism of improving outputs from living organisms. Small changes in the composition of the growth media can induce not only variation in the amount of specific compounds, but also the production of a completely different pattern of molecules [20,21,22]. Zeeck and co-workers coined the term “OSMAC” (one strain many compounds), which simply summarized the ability of single strains to produce different compounds when growing under different conditions [20]. While our understanding of the physiological and nutrient factors that pervade in the marine ecosystem is still partial at best, some clues have emerged to underpin early culture improvement studies. Screening of a panel of extracts from the marine fungus *Spicaria elegans* grown under 10 different culture conditions revealed a dramatic shift in the profile of secondary metabolites produced [23]. This included the isolation of the novel spicochalasin A, five new aspochalasins M–Q, and two known aspochalasins. Subjecting the marine-derived fungus *Ascotricha* sp. ZJ-M-5 to the OSMAC approach yielded three new caryophyllene derivatives and the known 1,3,6-trihydroxy-8-methylxanthone in Czapek Dox broth with or without Mg^2+^ [24]. Examples from other ecosystems are provided by the detailed work of Martin and co-workers, which repetitively showed how the phosphate concentration greatly influences the production of secondary metabolites in streptomycetes species [21]. They showed how cross-talk between global nutritional regulators has a great effect on both primary and secondary metabolites [25]. Similarly, also the carbon sources have been shown to greatly affect secondary metabolites production in a wide range of microorganisms [26]. Other easily accessible cultivation parameters can be modified, e.g., temperature, salinity, aeration, shape of the flasks, and these strategies led Zeeck and colleagues [20] to show that *A. ochraceus*, known to synthesize only aspinonene, was able to produce 15 additional metabolites when grown under different cultivation conditions. Similarly, applying the OSMAC approach three new compounds belonging to the rare class of 22-membered macrolacton polyketides were isolated from the bacterium *Streptomyces* sp. C34 [27]. The power of this approach was further reinforced by the work from Paranagama and colleagues [28], who were able to characterize six new secondary metabolites produced by the plant-associated fungus *Paraphaeosphaeria quadriseptata,* simply by changing the water used for the media preparation from tap water to distilled water. Although the exploration of bioactive compounds from the marine environment is gaining an increased interest in recent years [29], systematic studies aimed at testing the effect of different cultivation conditions on secondary metabolite production by microorganisms are scarce. Nevertheless, changing nutrient regimes, and media composition have been shown to influence secondary metabolites production also in marine microorganisms [30,31]. Notwithstanding these advances, there are limitations to the use of this approach, highlighted by the strain-specific variation observed in the quantity of metabolite production as well as the seemingly capricious behavior of fungi to alter metabolite profiles when re-cultured [32].

### 2.2. Challenging Microorganisms with External Cues

In addition to the cultivation parameters, external cues have been used to deviate cellular metabolism towards secondary metabolite production. For example, Christian and co-workers were able to isolate three new secondary metabolites, chaetoglobosin-510, -540, and -542, from cultures of the marine derivate fungus *Phomopsis asparagi* challenged with the F-actin inhibitor jasplakinolide [33]. Another example is the production of the potent antibiotic jadomycin by the bacterium *Streptomyces vanazuelae*. When cultured under physiologically favorable conditions in the absence of cellular stress, low amounts of jadomycin are produced. However, when cells are subjected to heat- or ethanol-shock, production of the compound is greatly increased [34]. Similarly, heat- or ethanol-stressed *S. hygroscopicus* cells produce a significantly higher amount of the antibiotic validamycin, with concentrations up to 13 g/L harvested [35,36]. More specific chemical cues have also been recently used. Another interesting line of research concerns the use of antibiotics, or molecules strictly related to them, to elicit the production of secondary metabolites. Studies in *Burkholderia thailandensis* showed that sub-lethal concentrations of trimethoprim served as a global activator of secondary metabolism by inducing at least five biosynthetic pathways, when present at sub-inhibitory concentrations [37]. Similarly, a series of molecules related to the synthetic antibiotic triclosan, were able to enhance the yield of secondary metabolites produced by *S. coelicolor* [38]. In a broader sense, these cues or chemicals have been shown to alter the secondary metabolite production in a wide range of streptomycetes strains, leading also to the identification of several novel compounds [39]. Together with the successful chemical elicitation of other novel secondary metabolites including lunalides A and B, oxylipins, cladochromes F and G, nygerone A, sphaerolone, dihydrosphaerolone, mutolide and pestalone [40], these examples clearly show how such stresses greatly influence the production of secondary metabolites, suggesting easy approaches to assess the biosynthetic ability in less explored microorganisms.

### 2.3. Synergistic Interplay among Microorganisms

Microorganisms live in complex communities in dynamic and constantly changing environments. In order to increase their fitness, microbes need to adapt to both the different environmental conditions and the presence of different competitive species. Communication between cells and between species has long been known to be central to the activation or suppression of key cellular metabolites, whereby individual cells can monitor group behavior and tailor transcriptional and translational activities accordingly. Based on this assumption, the interplay between strains of the same or different species has been used to enhance the production of known compounds and to discover new bioactive natural products [16,41,42,43,44,45]. Among marine microorganisms, the co-cultivation approach has been shown to represent a promising strategy to discovery new bioactive compounds. For example, a new benzophenone antibiotic, called pestalone, that exhibits potent antibacterial activity against drug-resistant bacteria, was isolated from a mixed fermentation of a deuteromycete (*Pestalotia* sp.) and an unidentified antibiotic-resistant marine bacterium [41]. Another successful example comes from the expression of the biosynthetic pathway for the production of four different diterpenoids, libertellenones A–D, which show varying levels of cytotoxicity against the HCT-116 human adenocarcinoma cell line. Libertellenones’ production was obtained by culturing the marine fungus *Libertella* sp. with a marine alpha-proteobacterium [46]. Similarly, the co-cultivation of two mangrove endophytic fungi led to the discovery of marinamides A and B [47], while Angell and co-workers showed that mixed bacterial culture isolated from ocean floor sediments produced blue pigment with antibiotic activity [48]. The pigment was characterized as the phenazine derivative pyocyanin, and it was produced by a *Pseudomonas aeruginosa* strains only when co-cultivated with a strain of *Enterobacter* sp. Finally, the coral bacterium *Bacillus amyloliquefaciens* GA40 stimulated production of lipopeptide antifungal metabolites when grown in the presence of *A. fumigatus* and *A. niger* [49]. The strength of this approach has further been shown in one of the first systematic studies conducted to investigate the activation of cryptic or silent BGCs in the model fungus *A. nidulans* during co-cultivation with different actinomycetes [44]. Among the tested actinomycetes, *S. rapamycinicus* was found to selectively activate two silent gene clusters. One of the gene clusters produced the aromatic polyketide orsellic acid, lecanoric acid, and the cathepsin K inhibitors F-9775A/F-9775B. Onaka and colleagues conducted an interesting study on the effect of mycolic-acid containing bacteria on the production of secondary metabolites by actinomycetes strains [43]. They could show that *Tsukamurella pulmonis* enhances the production of known metabolites in 54.5% of the tested actinomycetes strains and triggers the production of unknown compounds in 36.6% on the strains.

Although applicable only to cultivable microorganisms, the co-cultivation approaches represent a solid and promising strategy to discovery new bioactive metabolites. In this respect, the recent effort in the isolation of new marine microorganisms for the discovery of unknown secondary metabolites offers an invaluable platform to explore multiple cultivation conditions, external cues, and co-cultivation possibilities, capitalizing on the vast diversity hidden onto the marine environments (Table 1). While the underlying factors responsible for activation of silent BGCs during co-cultivation are likely to be species and combination specific, the signaling systems described in the following sections are likely to play some role.

**Table 1:** Methods and outcomes for activation of cryptic clusters.

Organism	Compound	Technique	Ref.
*A. ochraceus* (DSM-7428)	Aspinolides and Aspinonene/Aspyrone co-metabolites	Alteration of cultivation conditions	[50]
*Streptomyces* sp. strain C34	Chaxalactins A–C	Alteration of cultivation conditions	[27]
*Paraphaeosphaeria quadriseptata*	Cytosporones F–I; Quadriseptin A; 5′-Hydroxymonocillin III; Monocillin I and III; Aposphaerin B	Alteration of cultivation conditions	[28]
*Phomopsis asparagi*	Chaetoglobosin-510, -540, and -542	External cues	[33]
*Pestalotia* sp.	Pestalone	Co-culture	[41]
*Libertella* sp.	Libertellenones A–D	Co-culture	[46]
Uncharacterized endophytic fungi	Marinamide A–B	Co-culture	[47]
*Pectobacterium carotovorum*	Orange pigment	Quorum-sensing	[51]
*B. cepacia*	Enacyloxin Iia iso-enacyloxin IIa	Quorum-sensing	[52]
*B. thailandensis*	Novel thailandamide lactone variant	Mutation in transcription factor	[53,54]
*Streptomyces* sp.	Angucyclinone	Mutation in transcription factor	[55,56]
*S. coelicolor*	Uncharacterized antibacterial compound and pigment	Mutation in transcription factor	[57]
*S. orinoci*	Spectinabilin	Artificial promoters	[58]
*Saccharomonospora* sp. CNQ-490	Taromycin A	Artificial promoters	[4]
*A. nidulans*	Emodin, monodictyphenone, and F9775A/F9775B	Epigenetic mining	[59]
*S. lividans*	Blue-pigmented antibiotic actinorhodin	Ribsomomal Engineering	[60]

### 2.4. Microbial Signaling and Cryptic Clusters

Another class of compound that has been shown to elicit activation of BGCs is the quorum sensing class of signaling molecule. A key control mechanism for cellular physiology, virulence and antibiotic production, quorum sensing molecules are widespread among microbial organisms. The nature and chemical composition of these signal molecules can be quite diverse, with the paradigm class of signal for Gram negative organisms being the acyl-homoserine lactone (AHL) class [61]. First discovered in the marine symbiont *Vibrio fischeri*, AHL signaling systems have been identified in several marine organisms, suggesting a role in population dynamics within this ecosystem [62,63,64,65]. However, the nature of cell–cell signaling in marine sponges and other marine niches remains less well understood. Nevertheless, this signal based mechanism for control of cellular behavior has already been shown to coordinate antibiotic biosynthesis within producing bacteria, as seen with pyrrolnitrin production in a rhizospheric biocontrol strain of *Serratia plymuthica* [66], and with production of a polyketide antibiotic in *B. thailandensis* [67]. The quorum sensing controlled Evr transcriptional regulator was shown to activate a conserved cryptic pigment biosynthetic cluster and a novel phenomycin-like locus in the plant pathogen *Pectobacterium carotovorum* [51]. Intriguingly, synthase genes encoding AHL signal molecules have been identified adjacent to BGCs, e.g., indolmycin production in *Pseudoalteromonas luteoviolacea* [68], raising the possibility of a direct role in the activation of these BGCs [69]. Enacyloxin IIa and its stereoisomer designated iso-enacyloxin IIa were identified as metabolic products of a QS modulated cryptic gene cluster in *B. cepacia* complex (Bcc) isolates, in this case encoded in a genomic island [52]. The biosynthesis of carbapenem antibiotic and prodigiosin pigment in *Serratia* is also under quorum sensing control, a feature that has been used for the development of biosensors for QS production and interference [70]. In some cases, the regulation may be negative, with null mutation of the LuxR-type quorum sensing regulator system in *B. thailandensis* found to activate the thailandamide biosynthesis gene cluster, dramatically increasing thailandamide production, with an associated strong pigmentation [54]. Other classes of quorum sensing molecules have also been implicated in the production of natural products, and in many cases these tend to be more species specific than the broad spectrum AHL class. An example of this would be phenazine production in *P. aeruginosa*, which is controlled by the Pseudomonas Quinolone Signal (PQS) QS system [71]. Among Gram positive organisms, quorum sensing is typically orchestrated by auto-inducing peptides, while butenolide-type autoregulators have recently been reported to be involved in regulating antibiotic production in *Streptomyces* [72].

A core element of the synergism between organisms is the role of chemical messages or signal molecules in altering gene expression and secondary metabolite production of competing organisms. A major factor in shaping microbial populations, signal molecules produced by one organism have been shown to modulate phenotypes in species from other families or even kingdoms, in a form of communication or microbial diplomacy [73,74,75,76]. From an ecological perspective, the machinations of this interaction can be complex, and the terminology surrounding the actual role of these compounds has triggered some debate [77,78]. Notwithstanding this, in the context of natural product discovery, the ability of these signals to activate silent BGCs has significant potential [16]. Both PQS and its biological precursor HHQ have been shown to elicit phenotypic changes in Gram positive and fungal organisms, with staphyloxanthin production in *Staphylococcus aureus* being enhanced in the presence of the latter compound [75]. Netzker and colleagues described the interaction between the filamentous fungi *A. nidulans* and *A. fumigatus* with the soil bacterium *S. rapamycinicus* at the molecular level, highlighting the involvement of interkingdom communication in the activation of silent BGCs [16]. Indeed, antibiotics themselves are being recognized as a class of signal or cue involved in interkingdom communication with the “weapons or signal” debate receiving a lot of attention [79]. As already described here, antibiotics have been widely reported as elicitors of biosynthetic products, although the degree to which they can activate a broad spectrum of silent BGCs remains to be ascertained.

## 3. Semi-Synthetic and Molecular Activation of Silent BGCs

The advent of molecular biology and the exponential increase in associated technologies has provided new opportunities for improving natural product isolation and production in microorganisms. In many cases, engineering strains to circumvent the native regulatory systems that govern the expression of these energy expensive secondary metabolites can significantly enhance production and isolation. These technologies are currently being applied to organisms from many diverse ecosystems, with their implementation limited mainly by the genetic pliability of the target organism. Most widely applied to accessing novel metabolites from uncultivated soil bacteria, examples of the application of these technologies to marine associated organisms are less evident. Notwithstanding this, while the broad genetic diversity and largely uncharacterized genetic imprints from rare marine organisms may limit the applicability of these approaches to the marine ecosystem, the core technologies have significant potential. Indeed, the adaptation of these approaches for isolation and characterization of marine derived secondary metabolites may represent the next stage in the development of these technologies.

### 3.1. Ribosome and Polymerase Engineering

The regulation of BGC expression is likely to be complex in light of the metabolic burden placed on the cell following production. However, at the simplest level, one of the limitations to overexpression of antibiotics and natural products under culture conditions is the transcriptional and translational requirement on cells. Removing the perception of the burden has the effect of increasing production, while also changing the profile of compounds produced. Different from other “pathway specific” approaches, this generalized activation has been attributed to mutation at Lys-88 to either Glu or Arg in the ribosomal protein S12 of Streptomyces species, which enhances protein synthesis in stationary growth phase [80].

Ribosome engineering as a concept emerged from the finding of abundant quantities of the blue-pigmented antibiotic actinorhodin in a strain of *S. lividans* with an altered RpsL protein, a critical component of the A-site of the 30S ribosomal subunit involved in both tRNA selection [81]. This was in contrast to the majority of *S. lividans* strains which normally do not produce antibiotics due to the dormancy of their antibiotic biosynthesis genes. Possibly circumventing the need for binding by the alarmone ppGpp, targeting the *rpsL* gene in microbes has shown potential as an emerging method for the activation of silent gene clusters [60]. Screening for *rpsL* mutants is relatively simple, based on the premise that rifampicin selective pressure will result in drug-resistant mutants. Already, considerable success has been reported, enhancing the yield of secondary metabolites in a wide range of structural classes, including polyketides, macrolides, aminoglycosides, and nucleosides (reviewed in [60]). In one study, screening of 1068 actinomycetes isolated from soil, identified 6% of non-Streptomyces actinomycetes species and 43% of Streptomyces species acquiring the ability to synthesize antibacterials against *Staphylococcus aureus* after a selection step that generated spontaneous *rpsL* or *rpoB* mutations [82]. Importantly, not alone was antibiotic production increased, novel antibiotics were also elicited through this approach, suggesting a broader applicability for activation of silent BGCs [82].

*RpoB* mutations have been widely effective in activating silent and poorly expressed secondary metabolite-BGCs at the transcriptional level in *S. griseus*, *S. coelicolor*, and *S. erythraea* [60]. The introduction of the *rpoB* mutation S487L into a *Bacillus subtilis* strain resulted in cells that overproduced an aminosugar antibiotic, 3,3′-neotrehalosadiamine (NTD), the production of which is dormant in the wild-type strain, suggesting applicability in a wider spectrum of organisms [83]. The observation that several actinomycetes possess two *rpoB* genes, in contrast to the widely accepted consensus of the existence of a single RNA polymerase (RNAP) in bacteria, presents a new opportunity to develop this technology [60,84]. Two *rpoB* paralogs, *rpoB*(S) and *rpoB*(R), provide *Nonomuraea* sp. strain ATCC 39727 with two functionally distinct and developmentally regulated RNA polymerases. Heterologous expression of *rpoB*(R) in *S. lividans* resulted in activation of cryptic antibiotic biosynthesis, both in the wild type 1326 strain, and also in a relaxed (rel) mutant strain KO-421, unable to produce ppGpp [85]. The capacity for ectopic activation in heterologous hosts further enhances the feasibility of this technology for use in natural product biodiscovery.

### 3.2. Awakening the Activator

**Figure 3:**
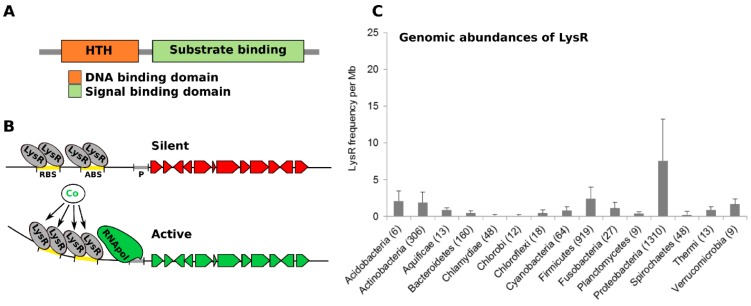
LysR regulation and cryptic clusters. (**A**) LysR transcriptional regulators are comprised of two domains, a DNA binding HTH domain typically found at the *N*-terminal, and a co-inducer or signal binding domain; (**B**) As with several other families of transcriptional regulators, LTTRs are signal responsive, present either in an inactive or sometimes repressive state on the promoters of biosynthetic genes in the absence of an environmental cue. Upon addition of the appropriate signal or co-inducer, conformational changes occur at the promoter eliciting activation of the target gene(s); (**C**) The frequency of genes encoding LTTR proteins is significantly higher among the genomes of families known to include well characterized bioactive producing organisms. The specificity with which these proteins regulate gene expression and their inactivity in the absence of an activating signal make them likely candidates for control of cryptic biosynthetic gene expression.

A recurring theme from the genomics based discovery of BGCs is the co-occurrence of genes encoding transcriptional regulatory proteins suggesting a programmable activation or repression of BGC expression. Many secondary metabolite BGCs contain one or more genes that encode transcription factors that potentially transcribe all the genes of the cluster. Of the BGCs characterized to date, a co-occurrence of the LysR-Type Transcriptional Regulator (LTTR) family with BGCs has been described for a diverse spectrum of bacterial organisms [86,87,88]. Known to control the expression of metabolic and virulence related functions in a broad spectrum of microbial species, these regulators have dual domains typically consisting of an *N*-terminal DNA binding domain and a *C*-terminal co-inducer or ligand binding domain (Figure 3A and [89]). Capable of binding to DNA in the absence of a signal, they nonetheless remain inactive and expression is not achieved. Upon interaction with a co-inducer ligand or substrate, a conformational change ensues, with the formation of tetrameric structures and subsequent activation of expression at target promoters (Figure 3B). The capacity to interact with promoters in the absence of signal is an interesting feature as it typically retains the promoter in the off-state, as is the case with silent BGCs. Therefore, identifying an activating signal has the potential to change the regulator to the on-state leading to activation of the BGC and production of the bioactive compound. In some cases, LysR proteins may act as repressors of expression whereby deactivation of the protein is required for elicitation of biosynthesis.

ThnI has been shown to be required for synthesis of the β-lactam antibiotic thienamycin in *Streptomyces cattleya* [87], while another LysR protein, designated ORF-L16, is encoded within the spinomycin biosynthetic cluster of *Saccharopolyspora spinosa* [88]. The oxazolomycin BGC of *S. albus* encodes the OzmR LysR transcriptional regulator [90]. As with many of the LysR proteins identified to date, the role of this protein in the synthesis of oxazolomycin A, a peptide-polyketide hybrid compound containing a unique spiro-linked β-lactone/γ-lactam, a 5-substituted oxazole ring, is unknown. However, the co-occurrence of LysR proteins within bioactive gene clusters strongly suggests a role in their transcriptional regulation. Intriguingly, LTTRs were found to be most abundant among the Actinobacteria, Proteobacteria and Firmicutes (Figure 3C), the three phyla that account for the majority of natural product biosynthetic potential [91]. A recent molecular phylogenetic analysis of LTTR proteins from the Proteobacterial pathogen *P. aeruginosa* revealed clustering of the LTTR repertoire into nine independent clades [92]. Importantly, clustering of either the DNA binding domain or the co-inducer binding domain resulted in conservation of the clades indicating that some degree of structural similarity may exist within the activating signal. Extending such an approach within the genomes of BGCs-rich organisms may yield clues on the activating signals that activate the LTTR regulated module, providing hints on the experimental approaches to follow to express the BGCs.

Of course, several other classes of transcriptional regulator have also been described in association with BGCs. Several reports describe the co-occurrence of *Streptomyces* antibiotic regulatory protein (SARP) family proteins with BGCs. These include the *aur1* polyketide gene cluster involved in biosynthesis of the angucycline-like antibiotic auricin in *S. aureofaciens* [93], ThnU which is required for cephamycin C biosynthesis in *S. cattleya* [87], as well as several other *Streptomyces* sp. related clusters [94,95,96,97]. The TetR and AraC families are also represented, with many regulatory systems co-occurring within the large BGCs [98]. In addition to the pathway specific activation of BGCs, some global regulators of secondary metabolite production have been identified. The cyclic AMP receptor protein (Crp), which is known to regulate catabolite repression in *Escherichia coli*, has recently been shown to be a global regulator for antibiotic production in *Streptomyces* [99]. In addition to increasing antibiotic production, overexpression of Crp in several *Streptomyces* species also led to the production of new metabolites, while deletion of this global regulator resulted in dramatic reduction of antibiotic production levels.

Identifying the signal or conditions which elicit activation of transcriptional regulators can be difficult. Where activation remains elusive, an alternative strategy has been pursued in organisms amenable to molecular genetic manipulation, whereby the promoter of the transcription factor can be replaced by an inducible promoter. This has the effect of artificially driving expression of the transcriptional regulator, which should in turn result in production of the associated natural product [80,100,101]. There are obvious limitations to this approach where (a) post-transcriptional or post-translational regulation occurs; (b) activation of the transcriptional regulator requires physical interaction with a co-inducing molecule (as with LysR proteins); or (c) not all biosynthetic genes are under the control of a common regulator (as with many large BGCs in which several transcriptional regulators from different classes are encoded). Notwithstanding this, the use of inducible promoters has potential for application in this field.

### 3.3. The Mutation Approach: Deleting the Suppressors

While positive regulation by transcriptional proteins is the dominant phenotype, repression is also a feature of many, whether intrinsic or state dependent. In this case, deletion or deactivation of these suppressor proteins has the potential to free silent BGCs from their locked in state and result in production of the natural compounds. Members of the LysR and TetR families of transcriptional regulator are known to possess suppressive activity and are one potential target for this approach. Null mutation of the *hexA* LysR from *Photorhabdus luminescens* led to a dramatic increase in the biosynthesis of small molecules [102]. *Bth_II1681* encodes for a LysR-type TF associated with the previously characterized thailandamide biosynthetic cluster of *B. thailandensis* [53,103], with mutations in this regulator resulting in the production of a novel thailandamide lactone variant [53,54]. An example TetR repression was shown by the null mutation of repressors within two silent gene clusters in *Streptomyces* sp*.* PGA64 and *S. ambofaciens*, which induced expression of the BGCs and resulted in the detectable production of the corresponding natural products—novel angucyclinone and already described kinamycins, respectively [55,56]. In another study, deleting a presumed pathway-specific regulatory gene (*scbR2*) that encodes a member of the c-butyrolactone receptor family of proteins and which lies in the *cpk* gene cluster of *S. coelicolor* A3(2) led to production of novel antibacterial activity (abCPK) and a yellow-pigmented secondary metabolite (yCPK) [57]. In some cases, repression of biosynthetic clusters involves both transcriptional and post-translational control, as seen with production of the cryptic orsellinic acid gene cluster in *A. nidulans*. Genetic assessment of *mvlA* mutants revealed the role of both itself and VeA (but not the VeA partner LaeA) in the suppression of the cryptic ors gene cluster producing orsellinic acid and its F9775 derivatives [104]. Adding to the complexity of this regulatory system was the involvement of histone 3 acetylation, suggesting the involvement of epigenetic control [104].

In a broader sense, deletion of cellular systems also has potential for activation of silent BGCs. An example of this is where protein destabilization has been targeted for deletion resulting in recovery of novel bioactive compounds from *A. nidulans*. Specifically, deleting the conserved eukaryotic *csnE*/CSN5 deneddylase subunit of the COP9 signalosome results in the activation of a previously silenced gene cluster comprising a polyketide synthase gene producing the antibiotic 2,4-dihydroxy-3-methyl-6-(2-oxopropyl)benzaldehyde (DHMBA) [105]. The highly conserved nature of this system among eukaryotic organisms underpins its potential as a broad spectrum approach for the activation of silent BGCs.

### 3.4. Artificial Promoters

While attention has focused on the manipulation of transcriptional activation of BGCs, another approach has been the insertion of inducible artificial promoters to drive expression of the silent genes. This generally takes advantage of the advances in molecular cloning technologies where suitable plasmid systems are generated in which structural biosynthetic genes can be cloned downstream of strong promoters, thus overcoming the cryptic native transcription network. Decoupling pathway expression from the complexity of native regulation circumvents the need for culture specific conditions and in some cases the laborious search for appropriate activating signals. In addition, production of the respective natural products can be controlled under defined culture conditions, ensuring downstream processing and stabilization prior to isolation. This has received a lot of attention in fungal systems where the transcriptional control of large clusters can be manifestly complex [18]. Described as refactoring, already this approach has met with some success, as with activation of the silent spectinabilin and taromycin A pathways from *S. orinoci* and *Saccharomonospora* sp. CNQ-490, respectively [4,58]. In the latter case, transformation-associated recombination (TAR) cloning was exploited to express a 67-kb nonribosomal peptide synthetase BGC from the marine actinomycete *Saccharomonospora* sp*.*, producing the dichlorinated lipopeptide antibiotic taromycin A in the model expression host *S. coelicolor* [4]. In another study, Luo and colleagues used a similar strategy to activate a cryptic BGC SGR810-815 from *S. griseus*, resulting in the production of three novel polycyclic tetramate macrolactams [106]. In what the authors of each study described as a “plug and play scaffold”, the respective host strains were engineered using a specific set of heterologous promoters that were functional in a heterologous host under the target culturing condition. More recently, a single genomic capture and expression vector for antibiotic production in *Bacillus subtilis* has been reported [107], while the pathway for production of alterochromide lipopeptides by *Pseudoalteromonas piscicida* JCM 20779 was heterologously expressed in *E. coli* utilizing native and *E. coli*-based T7 promoter sequences [108]. Heterologous expression of genes from the proposed aspyridone biosynthetic cluster from *A. nidulans* in the host *A. oryzae* led to the production of eight different compounds in addition to aspyridone A 1, one of the previously observed products [18]. Surprisingly, the previously accepted final product of the pathway, aspyridone B 2, was not detected [18]. Similarly, expression of silent gene clusters has also been achieved in *Streptomyces* using the strongly constitutive promoter ermE*p [109], while introduction of the transcriptional activator of balhimycin biosynthesis, the *bbr* gene from *Amycolatopsis balhimycina* (*bbrAba*) into *A. japonicum* also resulted in the production of an antibiotic-active compound [110]. More recently, a new high-performance heterologous fungal expression system was described in *Aspergillus terreus* [111]. Based on regulatory elements from the terrein gene cluster, the system was found to be particularly suitable for high level expression of polyketides in heterologous hosts. However, despite some successes, challenges remain and molecular-based methods utilizing heterologous expression systems continue to be limited by problems such as locating and cloning genes, difficulties with gene transformation and inactivation, and host incompatibilities [32].

### 3.5. Epigenetic Mining

Of course, while transcriptional control by regulatory proteins is key to unlocking the silent potential of BGCs, epigenetic control also needs to be addressed. Within the broad grouping of microorganisms, but particularly in the higher order organisms such as fungi, phosphorylation, acetylation, methylation, ubiquitination, ADP-ribosylation, and glycosylation are known to regulate gene expression [112]. Although this aspect of BGC control is relatively uncharacterized, there is evidence that modulating epigenetic control can enhance or activate production of natural products in fungal organisms [80]. Some early indication of the importance of global regulatory mechanisms came from the observation that the putative nuclear regulator LaeA controlled fungal secondary metabolite production [113]. Around the same time, the observation that published fungal genomes demonstrate a propensity for the positioning of many putative natural product BGCs in the distal regions of chromosomes was reported [114]. When we consider that these regions typically exist in a heterochromatin state, where gene expression requires epigenetic control, a role in the expression of silent gene clusters linked to natural product expression appeared likely.

Such was the case with disruption of the *hdaA* gene encoding an *A. nidulans* histone deacetylase (HDAC) leading to the transcriptional activation of genes involved in the biosynthesis of sterigmatocystin and penicillin [114], while deletion of the *cclA* gene encoding one of the eight COMPASS complex proteins, led to the activation of at least two silent gene clusters involved in the biosynthesis of emodin and the related compounds, monodictyphenone, and F9775A/F9775B [59]. This is not surprising given the analogous relationship between DNA supercoiling and microbial pathogenesis, suggesting an intrinsic role within the regulation of cellular physiology [115]. Deletion of *sumO* in *A. nidulans* caused a dramatic increase in asperthecin and a decrease of austinol/dehydroaustinol and sterigmatocystin production [116], while culturing in the presence of DNA methyltransferase (DNMT) or HDAC inhibitors led to isolation of novel compounds from several fungal organisms [32]. This latter observation shows the potential use for small-molecule epigenetic modifiers for accessing silent natural product pathways and enhancing the native production of fungal secondary metabolites. Furthermore, the success of this approach, with the added benefit of bypassing complex molecular manipulations and multifactorial culture based approaches, highlights the potential application of epigenetics to natural product discovery. Indeed, the multitude of chromatin modifications which have been found to regulate expression of secondary metabolites [117], and its potentially hierarchical position relative to transcriptional control, highlights the importance of understanding and exploiting this phenomenon for activation of silent secondary metabolism.

Epigenetics may also play a role in triggering the activation of silent fungal BGCs in response to co-cultivation with bacteria. As already discussed, contact with competing microbial organisms can elicit activation of secondary metabolite production, with interspecies and interkingdom communication receiving a lot of attention in the fields of ecology and microbial pathogenesis [75,76]. *S. rapamycinicus* triggered modification of fungal histones, eliciting production of the archetypal polyketide orsellinic acid and its derivatives in *A. nidulans*, following co-cultivation [118]. Deletion analysis of 36 of 40 acetyltransferases, including histone acetyltransferases (HATs) of *A. nidulans*, implicated the Saga/Ada complex which was also shown to play a major role in induction of other BGCs, such as sterigmatocystin, terrequinone, and penicillin [118].

## 4. Future Perspectives: Expanding the Natural Horizon

The potential for new product discovery following the implementation of emerging methodologies within the marine biodiscovery pipeline is an exciting one. The breadth of diversity that exists within BGCs (*i.e.*, one species many compounds, multiple BGCs feeding single natural products) already hints at the prospect of new structures with more potent and attractive activities, for the pharmaceutical, medical and industrial sectors. Crucial to the importance of the marine ecosystem is its already demonstrated potential to deliver on distinctive secondary metabolites when compared with other niches. Rather than simply finding the same compounds in a different environment, the marine ecosystem has delivered on unique features within secondary metabolites, as seen with the halogenation of many natural products. While rare in terrestrial organisms, halogenated secondary metabolites are common in the marine ecosystem, being produced by marine bacteria, algae and invertebrates [119,120,121,122]. Marine organisms appear to reflect the oceanic abundance of chloride and bromide in their secondary metabolites, producing most of the 4000 known natural organohalogens [120,122]. Other signatures that reflect the distinctive nature of the marine ecosystem are likely to manifest, underpinning the latent potential this ecosystem presents. Reaching this diversity, and our ability to culture the rare producers from the marine ecosystem, is the focus of ongoing research programmes such as the EU funded MaCuMBA (Marine Microorganisms: Cultivation Methods for improving their Biotechnological Applications) [123]. However, we do not need to rely on nature as a sole provider of this complexity, and the capacity for novel compounds to themselves act as new scaffolds or frameworks for synthetic or chemical modification provides yet more potential value to the chain of production. Already, the integration of synthetic biology and combinatorial chemistry approaches into the biodiscovery toolkit have led to significant improvements and advances in the area of natural product development.

### 4.1. Synthetic Biology

Parallel to the advances in biodiscovery and natural product chemistry have been the developments in synthetic biology, whereby synthesis and synthetic manipulation of natural products has superseded the need for biological production. Indeed, it has recently been proposed that synthetic biology is poised to reinvigorate interest in natural products as sources of new antibiotics [124,125,126]. Synthetic biology, described as the application of rational engineering processes to biological systems, has replaced the prior approach of random and laborious manipulation of genetic information encoded in biosynthetic genes. Once the biosynthetic pathways are understood, from the individual components to the sum of the parts, synthetic biology can format a production line in a targeted fashion to generate libraries of structurally similar compounds which can then be screened for favorable activities. In a form of directed evolution, it can significant accelerate the process of bio-discovery, and in just a few years the synthetic biology approach and concepts have moved from an avant-garde concept to a solid and powerful reality [127]. In addition, genetic manipulation of producing organisms or heterologous hosts has the demonstrated potential to prime the metabolic network for increased production of sought after secondary metabolites [128]. Described in some studies as a form of “plug and play”, synthetic biology will ultimately allow us to integrate newly sequenced pathways into pre-engineered microbial hosts primed for the overproduction of compounds synthesized from specific pathway classes [124].

From a synthetic biology perspective, marine BGCs represent an almost inexhaustible resource for the synthesis of new metabolites [129,130,131]. Most of the BGCs are characterized by a high degree of modularity (e.g., PKS, NRPS). This characteristic allows both the refactoring of genes belonging to a specific cluster and combination of specific modules among clusters. Such approaches can lead to both an increase in yield of a specific metabolite and to the synthesis on new non-native compounds [124,132]. One outstanding example is the pioneering work of Keasling and co-authors that, combining plant and bacteria derived genes, were able to engineer both *E. coli* and *S. cerevisiae* to produce amorphadiene and artemisinic acid, precursors of the potent antimalaria drug artemisinin extracted with laborious procedures and low yields from the plant *Artemisia annua* [133,134,135,136]. The power of this approach was recently shown in a study that led to the discovery of 74 novel compounds produced using a combinatorial genetic approach in yeast [137]. Interestingly, >75% of the identified molecules have not been described previously.

The great phylogenetic diversity of marine microorganisms together with the astonishing biosynthetic diversity emerged from the recent development of the sequencing technology are already giving new hope for the identification of new valuable chemical products. The biological features of the known biosynthetic systems represent rich toolbox that can produce an inestimable chemical diversity [124]. The synthetic biology approach might present in the near future the necessary keystone to explore such diversity. However, the undoubted potential of this technology currently comes at a price, and the complexity of BGCs [138] ensures that synthetic biology will not replace the biological approach, at least for the foreseeable future. Refactoring BGCs via a synthetic biology approach is highly resource intensive, bearing in mind the large size of most secondary metabolic gene clusters (multiple genes at a typical length of 20–150 kb). Taking into account current gene synthesis costs, the technical challenges involved in homologous recombination based assembly of gene clusters, and the likely requirement for generating multiple cluster variants, the cost per compound could be as high as $50K USD or more per compound [139]. Even where the economic limitations to synthetic biology are overcome, as they undoubtedly will in time, further challenges remain. The modular nature of many BGCs means that heterologous expression and refactoring is limited by the compatibility of reconstituted systems, something that cannot be predicted [140]. Additional bottlenecks with regards to heterologous expression and product isolation persist, in spite of marked advances in natural product isolation. These bottlenecks, which include rare codon usage, incompatible host machinery, and low titres, are likely to be reinforced with the activation of silent gene clusters from rare and previously uncultured organisms. However, the place of synthetic biology in the toolkit of marine biodiscovery is an important one, and future developments in this field will contribute significantly to advances in natural product discovery.

### 4.2. Combinatorial Chemistry

Secondary metabolites are excellent candidates for use in combinatorial chemistry due to their modular nature [141]. The fact that these compounds are often synthesized as polymer backbones that are subsequently diversified greatly via the actions of tailoring enzymes makes them pliable to modification [142]. This has led researchers to integrate the biological power of natural products with the synthetic capacity of organic chemistry, with technologies ranging from the combinatorial total synthesis of analogues to the exploration of natural product scaffolds and the design of completely unnatural molecules that resemble natural products in their molecular characteristics [143]. When applied to silent BGCs, and the derivatization of the as yet uncharacterized natural products, additional challenges may be faced. Currently, the option exists to use some natural products as building blocks or even scaffolds upon which to generate new and biologically distinct entities. The chemistry is based on known modalities, with optimized chemical reactions tailored to known activities. What happens when the building block is not recognized or does not fit the structural classes that have preceded it? Many classes of rare producing marine organisms have yet to be cultured, or their natural products isolated, with the result that previously unseen paradigms may emerge. The same applies to the synthesis of natural product analogues. While the synthesis programmes are well established within the pharmaceutical industry, structural enigmas may present themselves when the biosynthetic potential of the silent gene clusters are awakened. Furthermore, while the design of combinatorial libraries has been driven by the intent to generate a high degree of structural diversity within a library, future design will need to be guided not only by structural descriptors, but also by descriptors of biological activity [144]. This will be particularly relevant when we consider the dearth of information surrounding the structural basis of the natural products emanating from marine derived silent gene clusters.

### 4.3. Mining the Awakened Metabolome

Natural product biodiscovery in general has been greatly facilitated by advances in metabolomics [145,146], whereby differential metabolite profiles have shed light on the identification of bioactive natural products, which have then been structurally characterized by associated technologies such as multidimensional NMR [37]. The ability to measure the entire complement of metabolites within microorganisms provides us with a more definitive outcome or measure of bioactive production when compared with similar genetics based approaches which measure potential rather than the final product. Goulitquer and colleagues recently reviewed the application of mass spectrometry metabolomics profiling for functional elucidation in marine organisms and ecosystems [147]. Describing the spatio-heterogeneity observed from multiple studies as a form of “genome in motion”, they describe the importance of developing technologies that enable dynamic analysis of the metabolic flux that exists in the marine and other ecosystems. More recently, Romano and co-workers used ultra-high resolution mass spectrometry to investigate the exo-metabolome of marine *Pseudovibrio* sp*.* grown under phosphate limiting and surplus conditions [22]. They uncovered an unexpectedly large and diverse exo-metabolome that was greatly influenced by phosphate addition. Sarkar and colleagues applied metabolomics analysis to monitor secondary metabolite production in *A. nidulans* grown under various nutrient limitation conditions, thus identifying several polyphenolic compounds as well as a novel prenylated benzophenone derivative designated as preshamixanthone [148]. In another study, Forseth and co-workers identified cryptic products of the gliotoxin gene cluster using NMR-based comparative metabolomics [149]. Using differential analysis by 2D NMR spectroscopy (DANS) of metabolite extracts derived from *gli* knock-out and wild-type *A. fumigatus* strains, they were able to establish a detailed inventory of *gli*-dependent metabolites, all of which provide an insight into the biosynthesis of this natural product. In addition to targeted analysis of producing strains, general strategies for metabolomics-based prioritization of microorganisms for natural product research have been proposed [150]. With parallel advances in analytical technologies such as nano-spray desorption electrospray ionization (nanoDESI) mass spectrometry and MALDI-TOF-IMS, the application of metabolomics for the high-throughput and unbiased identification of cryptic secondary metabolites is certain to become a significant component of the natural product biodiscovery toolkit [145].

## 5. Conclusion: Is There Something We Do Not Know?

Great progress has been made in both the identification of marine BGCs and in the characterization of their diversity and distribution. Already studies are showing that several important natural products previously attributed to marine sponges are actually produced by the marine sponge microbiota. For example, the bioactive metabolites polytheonamide A, polytheonamide B, nazumamide A, konbamide and keramamide D, previously attributed to the marine sponge *Theonella swinhoei*, were shown to be produced by uncultivated “*Entotheonella* spp.” [151]. The huge diversity within the marine microbial biosynthetic repertoire of natural products is well established, the challenge remains, however, to fully exploit and harness it. The tools described in this review are part of the answer, driving activation and synthesis of new molecular entities. However, it is reasonable to consider that, despite the explosion in genome sequencing and the parallel developments in predictive programmes, there remains a wealth of genetic information that has yet to be visualized and, perhaps, new paradigms for sequence analysis that have yet to be encountered [152]. The available predictive platforms (e.g., AntiSMASH, BAGEL3, and SMURF) have equipped the research community with the tools for identifying known class of BGCs, and will still have a leading role in the screening for new BGCs in newly sequenced organisms [69,153,154,155,156,157]. However, we must consider the potential for marine BGCs encoding previously unforeseen structures, encoded in new cluster arrangements, with enough anomalies that current predictive software cannot recognize. These unknown unknowns may be the next step in natural product discovery. Of course, that may present its own challenges with respect to standardization and nomenclature, but that is a hurdle that will no doubt be surmounted.
